# Survivors of Hell: Resilience Amongst Unaccompanied Minor Refugees and Implications for Treatment- a Narrative Review

**DOI:** 10.1007/s40653-021-00385-7

**Published:** 2021-07-22

**Authors:** Irene Mateos Rodriguez, Veronika Dobler

**Affiliations:** Department of Psychiatry, University of Cambridge, Cambridge, UK

**Keywords:** Refugees, Unaccompanied minors, Resilience, Connectedness, Relationships, MHPSS

## Abstract

Approximately half of the world’s displaced population are children and a significant proportion of these are unaccompanied asylum-seeking children (UASC). UASC are the most vulnerable of all refugee populations. Up to 90% of UASC have been subjected to exploitation, including trafficking or being drafted into military groups. Having survived the perils of flight, UASC are confronted with continued challenges, including the uncertainties regarding their residential rights in host counties. Unsurprisingly, the prevalence of mental health problems is higher amongst UASC than in any other refugee group. Yet, Mental Health and Psychosocial Support (MHPSS) appears to neither reach nor engage UASC. This begs for re-examination of what and how MHPSS is offered. Despite high levels of adversity, UASC often have considerable resilience, and make remarkable recoveries. However, literature exploring their resources or their own views of what helps or hinders recovery is scarce. In this narrative review, we explore individual and systemic factors promoting recovery in UASC. We consider theoretical understanding of resilience, emerging data and user perspectives. From these we deduct four areas of resilience in UASC: *Individual factors* – prosocial behaviour, problem-solving skills; *Lifetime relationships* – positive early family relationships, connection with family and country of origin, positive peer and adult relationships in host country; *Acculturation –* integration of own and new culture, positive relationships with prosocial institutions; *Care arrangements –* supported but less restrictive living arrangements. We suggest, MHPSS may need to focus on enhancing social networks, including connectedness with positive relationships in the home-country, life-history work with a focus on resource-building experiences in addition to trauma, and promoting integration of old and new cultural values. We also conclude that despite growing data, there is a gap in both eliciting user perspectives and understanding adaptive resources, especially those emerging during early development and within their cultural setting.

**Supplementary Information:**

The online version contains supplementary material available at 10.1007/s40653-021-00385-7.

## Introduction

### Unaccompanied Minors and Prevalence of Mental Health Problems

By the end of 2019, 79.5 million people were forcibly displaced worldwide (UNHCR, 2019). Between 2015 and 2016, 1.3 million refugees were seeking asylum in Europe, of which 32% were minors (Eurostat, 2018; Mitra & Hodes, 2019a). In 2015, there were 90,000 applications for asylum in European countries from unaccompanied minors, and 63,300 applications were made in 2016 (Eurostat, 2018; Mitra & Hodes, 2019a). Individuals under 18 years who have been separated from their parents and are asylum seekers, recognised refugees, or other displaced persons are referred to as ‘unaccompanied minors’, ‘separated from their parents children’ or unaccompanied asylum-seeking children (UASC) (Bean et al., 2007). This review will focus on unaccompanied minor refugees, rather than other groups of immigrant children. Under the United Nations High Commission for Refugees (UNHCR), the host country is bound to offer UASC legal support, housing, education, care, support, and protection (UNHCR, 2018).

The prevalence of mental and physical health problems amongst refugee children and adolescents is significantly higher than in the general population (El Baba & Colucci, 2018; Hodes et al., 2008; Jakobsen et al., 2014; Keles et al., 2016; Sleijpen et al., 2017). Prevalence of mental health problems in UASC, however, is higher than in children seeking asylum with their families, or children who are not from refugee or asylum-seeking backgrounds (Huemer et al., 2009), and it is higher than in unaccompanied children who are greeted by family on arrival to the new country (Van der Veer & Van Waning, 2004). Various studies suggested that between 41-69% of UASC report mental health problems (Bronstein & Montgomery, 2011; Jakobsen et al., 2014). Müller et al. (2019) reported that 64% of UASC meet diagnostic criteria for post-traumatic stress disorder (PTSD) (Müller et al., 2019) and female UASC may be more affected by post-traumatic stress symptoms and depressive symptoms than males (Mohwinkel et al., 2018).

### Cumulative Causes of Mental Health Problems in UASC

The factors associated with the higher prevalence of mental health difficulties amongst UASC have been divided into pre-, during, and post-migration factors; and these are influenced by individual, family, community, and societal risk and protective factors (Fazel & Betancourt, 2018; Müller et al., 2019). Overall, socio-demographic and post-migration factors, such as lack of social support, experience of discrimination, or lack of everyday resources, were predictors for mental health symptoms in both accompanied minors and UASC (Müller et al., 2019). Exposure to trauma pre-, during, or post-migration has also been strongly associated with the higher prevalence of mental health problems in all groups. For example, Müller et al. (2019) suggested that traumatic experiences accounted for 10% of the variance for anxiety and 23.5% for post-traumatic stress symptoms. Notably, UASC reported significantly more traumatic experiences than accompanied minors, even when controlling for age (Müller et al., 2019). Pre-migration factors may include trauma not related to the displacement such as sexual abuse, developmental challenges, or pre-existing mental health problems, as well as traumatic events that triggered the flight (recruitment as child soldier, threat of family by militia or war). Migration-related trauma includes political violence, combat situations with serious accident or injury, sudden/violent loss of family and loved ones, traumatic bereavements, and adversities during flight including lack of food or water for several days, being stabbed/attacked/shot/hurt badly (Hodes et al., 2008; Pfefferbaum et al., 1999), with female UASC being at higher risk of sexual exploitation and abuse during the flight (Fazel & Betancourt, 2018; Mohwinkel et al., 2018). Post-migration factors include acculturative hassles (the struggles of adapting to a new culture), financial, housing and employment insecurities, education and social life disrupted, lack of social support, experience of discrimination, limited access to everyday resources, and ongoing uncertainty and scrutiny in the asylum-seeking process, the threat of being deported back to their country, or the fear of having to live illegally to prevent deportation (Allsopp & Chase, 2019; El Baba & Colucci, 2018; Bronstein & Montgomery, 2011; Fazel & Betancourt, 2018; Hodes et al., 2008; Jakobsen et al., 2014; Mitra & Hodes, 2019b; Müller et al., 2019; Trickey et al., 2012).

### Provision and Access to Care in Host Countries

The high prevalence of mental health problems amongst UASC puts pressure on the host country to address these needs through Mental Health and Psychosocial support (MHPSS). When exploring access to MHPSS, cumulative data from various European countries suggest, that, while almost 60% of UASC perceive a need for psychological support, only 12-36% of these actually access mental health services (Bean et al., 2006; Michelson & Sclare, 2009; Sanchez-Cao et al., 2013; von Werthern et al., 2019). Even if referrals are made, UASCS have high rates of missed appointments, e.g. of 33% according to Michelson and Sclare (2009) which is double that of accompanied minors (Michelson & Sclare, 2009). At the same time, there appears to be significant proportion of UASC presenting in emergency units with self-harm and suicidal behaviour (Ramel et al., 2015). Taken together, these findings suggest there may be important barriers to accessing mental health services. This is likely to be the result of a combination of factors, including language barriers, the high frequency of relocation (therefore difficulties registering with local health services) (Ciaccia & John, 2016; Sanchez-Cao et al., 2013; von Werthern et al., 2019); lack of knowledge about available services, both the Children and the Adolescent Mental Health Services (CAMHS) and the free/charity services; the requirements for access, for example proof of residency or birth certificates that might not be available or they may be fearful of showing (Ciaccia & John, 2016). There may also be misconceptions about the role of services (worries about confidentiality, services being associated with police or home office). Referrals to mental health services are usually made by social services (Bean et al., 2006; Michelson & Sclare, 2009). However, visits of social workers or support staff may be infrequent with professionals possibly not registering the degree of distress experienced by UASC. Further, UASC may have generally lost trust that adults or other new people may be able to help, as result of their previous encounters with individuals turning out to be smugglers, traffickers, militia or perpetrators of other sorts (Ciaccia & John, 2016). This is coupled with varied cultural understandings of mental health among the UASC (Ciaccia & John, 2016; Majumder, 2016; Sanchez-Cao et al., 2013). Majumder (2019) explored the understandings of mental health in a group of UASC from the Middle East and Africa (Majumder, 2019), living in the UK. The word *madness* and being *locked up* was prevalent in their discourse, as was the fear of being rejected and abandoned by society. These findings highlight the stigma that UASC associate with mental health and associated services which, although partly attributed to the perceptions of mental health problems in their own cultures, is also fuelled by the fear of harassment, intolerance from local communities, and perceptions of not being accepted by the host country (Delago et al., 2005; Fazel et al., 2005).

Even once access to CAMHS has been facilitated, engaging and retaining UASC in treatment remains a challenge (Sanchez-Cao et al., 2013). Majumder (2019) explored the experiences of UASC enrolled in CAMHS in London, and their carers. UASC described their expectation of patient-doctor relationships of professionals acting instructively, with the UASC adopting more passive roles (Majumder, 2019; Majumder et al., 2019). The gender and the ethnicity of the therapist were also described as barriers. For example, young men found it hard to open up to women therapists for fear of over-burdening them; and therapists of different ethnicities were described as ‘suspicious’. Therapy felt like regressing into their past experiences, rather than dealing with the difficulties they were facing in their present lives. Other studies have described the lack of cultural sensitivity amongst medical professionals (Kohlbry, 2011) or the inappropriate use of interpreters (Davies & Webb, 2000) as barriers in therapy. UASC might also be more familiar with other forms of healing, such as traditional healers, elders and other important providers of care. The host country/community might be limited by their own cultural understanding of mental health, and not feel able to offer types of healing that feel more accessible to UASC. Therefore, stigma, fear, culturally-informed understanding of mental health problems and interventions, as well as cultural insensitivity by providers act as barriers to accessing and engaging with mental health services in host countries.

In addition, both trauma-focused cognitive behavioural therapy (TF-CBT) and narrative exposure therapy (NET) have significant evidence showing their effect in reducing psychological distress amongst children and adolescents who have experiences trauma, or with a diagnosis of PTSD (Gutermann et al., 2016; Morina et al., 2016; Peltonen & Kangaslampi, 2019; Schaal et al., 2009). This effect has been observed compared to treatment as usual, a waiting-list group, or counselling alone, and across cultures, and it is maintained over time. However, there is a lack of representative studies and focused attention on UASC specifically (Morina et al., 2016; Neuner et al., 2018; Onyut et al., 2005; Ruf et al., 2010) or, when UASC where differentiated from other groups, effectiveness of interventions was found to be limited (Oppedal et al., 2019).

In summary, mental health problems in UASC are highly prevalent; however, only a fraction access MHPSS, and of these, only two thirds remain engaged. Further, even where potential treatments may be available, there is a rarity of studies focusing on UASC to prove effectiveness for this group. Therefore, there is a need for further investigation of how best to engage and support these highly vulnerable young people.

### Do We Need to Refocus?

There are clearly considerable challenges for UASC in accessing and/or accepting MHPSS support which warrant further exploration of what might make interventions more accessible, acceptable and adapted to their perceived needs. Nonetheless, it is worth highlighting that many of these youngsters are survivors against all odds. Despite the extent of their traumatic experiences, not all of them develop mental health problems, and many of those who do, recover to a good functional level (Keles et al., 2018). This suggests that UASC may bring considerable resilience and resources that may form a foundation for, and facilitate their successful journeys. Understanding their resilience and resources may help to refocus interventions for UASC. In this narrative review, we first consider literature on resilience-promoting factors in child and adolescent populations generally, and then explore resources and resilience factors that support recovery and integration for UASC more specifically. We do this by examining recent quantitative data as well as qualitative narratives and views of UASC populations accumulated over time (for research strategy see Supplementary materials).

## The Review on Resilience and Unaccompanied Minors

### Defining Resilience

When resilience first emerged as a concept, it was described as the ability to ‘do well’ despite adversity or risk, and this ability was thought to be a more or less static feature inherent to a particular individual (Masten, 2011). More recently, however, this definition has evolved to capture the dynamic mediating or moderating contribution of multiple ‘resilience factors’ (RFs) to positive development despite experiences of significant adversity or trauma (Bonanno & Diminich, 2013; Masten, 2011). The positive adaptation may not necessarily affect all functional domains, but may apply to one or more specific parts of life (Bonanno & Diminich, 2013), and it can co-exist with mental health difficulties (Bonanno, 2012; Masten, 2011). In fact, mental health difficulties may be part of the process of adaptation to abnormal circumstances (Papadopoulos, 1999). Resilience is also thought to be culturally shaped (Tummala-Narra, 2007; Ungar, 2006), as suggested by cross-cultural data looking into young adults across eleven different sites around the world, where resilience is influenced by a range of individual, relational, community, cultural, and contextual factors (Ungar et al., 2008). Resilience may therefore, be understood as cumulative intra-individual traits and/or contextual factors that promote physical or mental well-being in the presence of adversity.

### Resilience Factors in Children and Adolescents

There is extensive literature on resilience in children and adolescents who have suffered from childhood trauma (Fritz et al., 2018). Generally speaking, it is agreed that RFs are distributed across a multilevel framework, including intra-individual, micro- and macro environmental levels (Cicchetti, 2010). A systematic review conducted by Fritz et al. (2018) explored which factors across the various domains were associated with resilience in the context of early childhood adversity. Adversity included a range of traumatic and stressful experiences that are associated with increased risk of developing mental health problems such as loss of a significant person, discord within the family, poor parenting, traumatic life events or tragedy, childhood abuse/maltreatment or neglect (Fritz et al., 2018). In this context, stable self-concept and ability to self-regulate along with cognitive flexibility, the ability to re-appraise and low rumination, low impulsivity and low aggression emerged as intrapersonal RFs. Amongst the interpersonal factors, the quality of past and present relationships, such as secure attachments to family members and the sense of connectedness (past, present, with peer, family, or societal), were supported. RFs of the wider social ecology included high parental involvement and positive parenting, immediate family support, extended family support, family cohesion, a positive family climate and high community-level social support (Fritz et al., 2018). Across the domains, factors that increase adaptability (e.g. cognitive flexibility) and factors that promote positive social interactions, including formation and maintaining relationships, across the individual to macro ecological levels, appear pertinent to resilience in the face of adversity.

Overall, these findings highlight a central role for factors that promote positive relationships in fostering resilience amongst children and adolescents. However, these factors have been mainly drawn from studies conducted in Western cultures (USA, Canada, Australia, and Israel), and therefore the extent to which they mirror the ranges of cultures that constitute the current refugee population in Europe is undetermined.

An importance difference between the resilience literature of childhood adversity and that of refugee children, is the nature of the trauma. Many UASC have experienced multiple types of trauma, and are thus poly-victims, which underscores the diversity of this group. However, the trauma in childhood adversity studies is largely associated with care-giver perpetrated attachment trauma, whereas amongst refugee children, despite multiple trauma, these are more likely associated with contextual/circumstantial trauma, on the background of preserved attachment relationships. This distinction in the type and number of traumatic experiences may result in different resilience factors, and thus warrants future exploration. In addition to the type and number of trauma, adversity factors that are specific to the refugee populations include having to navigate high levels of uncertainty, cultural diversity and adaptation to a foreign country. More specifically for UASC is the physical absence of trusted carers throughout their migration journey and during the acculturation process. While it is likely that both intrapersonal and interpersonal resources ultimately promote resilience in the host country, it is important to determine how exactly relationships and other contextual factors are perceived as helpful in promoting recovery and well-being in this group.

### Resilience Factors in UASC That Promote Recovery and Adaptation in The Host Country/Postflight

Children and adolescents have long been victims of war and trauma. There is probably an equally long history of children alone or separated from their families seeking refuge in foreign countries. The narratives of children who fled during or after the second world war offer one well-documented source of retrospective assessments of experiences of unaccompanied minors after arrival in the host country (Freud & Dann, 1951; Moskovitz, 1985; Valent, 1998). Moskovitz (1985), for example, explored the narratives of four children who arrived in the United Kingdom following the Holocaust. They belonged to a group of 300 children rescued from Concentration Camps, who arrived as UASC in Windemere. On arrival these children varied in age, background, and experiences of care prior to being subject to the atrocities of the Nazi regime. However, they had all seen the unspeakable horrors of the concentration camps, severe interpersonal trauma and major transgression of moral boundaries in a situation with no hope for escape. They had been subject to hunger and neglect. Their lives had been governed by survival mode. They had all arrived unaccompanied by a familiar carer and without knowledge of language, and total uncertainty with respect to their future. The themes emerging from their narratives included their longing for connectedness with a parental figure (whether biological or adoptive) and relatedly, their desire to be appealing to adults in order to form these connections. Further themes included their immense adaptability, in addition to themes of assertiveness and the importance of self-agency and independence. In their retrospective assessment, the adult survivors recollect the importance of being allowed freedom with little restrictions (Moskovitz, 1985). They also recount the crucial role of peer relationships with similar lived experiences, which enabled talking about the past in their own terms (Moskovitz, 1985; Werner, 2012), using humour and altruism as defence mechanisms (Werner, 2012). They further reported drawing strength from recounting happy early memories as well as secure attachments with their primary caregiver early on in life (Werner, 2012). While there are clearly many differences and significant limitations to the comparison, there are also parallels with UASC currently arriving in Europe, including direct or indirect experience of extreme trauma at the hands of organised perpetrators and the unaccompanied arrival and resettlement in a foreign country, but also the formation of some stable relationships during earlier childhood years. This is a key difference with the literature on resilience and early childhood adversity.

#### Resilience Factors in UASC – Current Literature

Although the literature on resilience in UASC is thin, a number of studies have focused on resilience in UASC recently arriving in Europe. In keeping with a multilevel framework, these can be summarised under the headings of individual factors, and factors relating to the past and present social ecology.

*Individual factors.* Review papers exploring the sources of resilience amongst individual UASC across the literature have identified a range of important factors. These include high cognitive function, adaptive problem-solving skills, easy temperament and positive self-esteem, individual faith in higher power or religious orientation, and conviction in needing to migrate (Carlson et al., 2012; Ehntholt & Yule, 2006; Nardone & Correa-Velez, 2016). Additionally, qualitative studies suggest that the capacity to create some form of meaning out of one’s adverse experience whilst appearing ordinary (Carlson et al., 2012; Goodman, 2004), and the recognition of self-agency upon completing the migration journey successfully (Nardone & Correa-Velez, 2016) are perceived as important features promoting resilience amongst UASC settled in new countries.

*Family relationships, peers and continuing bonds with own culture.* Qualitative studies exploring the narratives of UASC living in Western countries have stressed the importance of maintaining ties with families and/or extended families post-migration, whether in person or via technology (Chase et al., 2008; Ehntholt & Yule, 2006; Nardone & Correa-Velez, 2016). However, the potential for resilience through maintaining connection with families appears to be influenced by the connectedness within the family prior to migration, rather than by contact alone (Sierau et al., 2019; Sleijpen et al., 2016). The importance of these social support networks and their association with mental health problems was assessed by Sierau et al. (2019). They found that in a sample of 105 male UASC, from Afghanistan and Syria in Germany, using self-report measures, those individuals maintaining contact with families, via phone or social media, showed an increased ability to form new relationships with peers and other adults in the host country, in comparison to those who had no contact (Sierau et al., 2019). Maintaining this contact with families was also associated with, but did not moderate, lower scores on PTSD, depression, and anxiety scales (Sierau et al., 2019). Maintaining family relationships, especially within already connected families, is likely to provide an important psychological and practical resource that may mitigate against development of mental disorders, and thus facilitate adaptation and new relationships. This also appears to underpin a more stable self-concept with a sense of intra-individual continuity, inclusive of their past and present lives and cultural identities (Sleijpen et al., 2016; Southwick et al., 2014). Maintaining connections to one’s own culture of origin appears to also attenuate the distress and uncertainty relating to the disappearance, hurt, or death of loved ones in the midst and aftermath of war (Southwick et al., 2014).

Forming and maintaining new peer relationships has emerged as a further source of resilience amongst UASC migrating from many regions that include middle eastern countries, African areas of conflict, central/south American countries, and areas of the Kosovo conflict as well as Western countries including the USA, Australia, Belgium, UK, Spain, France and others (Demazure et al., 2018; Mels et al., 2008; Sujoldžić et al., 2003). A mixed-method study interviewing twelve UASC mostly from middle eastern countries in Belgium found that friendships formed with peers from similar ethnic backgrounds, with similar flight experiences, or in similar stages of the asylum process acted as ‘bridge builders’ that facilitate the process of acculturation (Mels et al., 2008). Close relationships with children at school and other adults (e.g. caregiver, guardian, teacher, or social worker), and prosocial institutions (e.g. schools or churches) have also been described as sources of support for, and have been associated with lower mental health problems amongst UASC (Chase et al., 2008; Ehntholt & Yule, 2006; Mels et al., 2008). In summary, relationships that enable individuals to maintain a continuous bond with individuals within their own culture, as well as forming new relationships with peers and adults from the host country, are likely to constitute important RFs amongst UASC.

*Process of acculturation: time and continuity.* Acculturation, as described at the beginning of the paper, refers to the process of adapting to a new culture when growing up in a bi/multicultural context (Oppedal & Toppelberg, 2016). The process involves understanding ways of thinking and behaving in the new culture, as well as negotiating the pressures from their own culture to maintain values and traditions (Oppedal & Toppelberg, 2016). The ability of UASC to adapt to their new culture has been found to be associated with increased resilience (Keles et al., 2016, 2018; Oppedal & Toppelberg, 2016). Keles et al. (2018) followed over 900 UASC in Norway over four years and found that having been in the country for at least four years was associated with higher scores on a cultural competence construct, and cultural competence was associated with increased resilience over time (Keles et al., 2018). Interestingly, interviews from thirty-two UASC from different countries in Ireland found that being able to continue their religious practice in Ireland, and meeting people from their own cultures in Ireland, including sharing their own language, and their home-country foods and films, facilitated the process of acculturation (Ní Raghallaigh & Sirriyeh, 2015). Coupled with this, embracing aspects of the local cultures, such as, language or styles of dressing, allows for this bicultural identity to develop (Miller & Kerlow-Myers, 2009). Overall, for the UASC, acculturation appears best achieved by maintaining both a continued connection with their own culture, as well as embracing the local culture. This process appears to be facilitated by the passage of time, as well as local and individual resources. A positive acculturation is associated with increased resilience (Keles et al., 2018).

*Care arrangements: type of placement*. Upon arrival in a country, UASC undergo an asylum process during which, and perhaps beyond, they would be placed in some form of care, as is mandatory under the Human Rights International Law (UNHCR, 2019). A number of quantitative studies have looked at the prevalence of psychological distress in different care arrangements. Jakobsen and colleagues found that a group of 138 male UASC aged between 15 and 18, in Norway from the Middle East and Northern Africa were placed in either reception centres for adults, for youth, or in specialised orphanages (Jakobsen et al., 2017). Reception centres for adults had low levels of support and care, where refugees were left to themselves to buy and cook their own meals, and there was no staff around for advice. Whereas, in reception centres for youth meals were cooked by the staff, they had language classes, recreational activities, and received individualised support and medical care, and the staff was present 24/7. The specialised orphanages were considered supportive living arrangements. At fifteen and twenty-six months, those in reception centres for adults scored higher on psychological distress and PTSD symptoms scales, compared to those in supportive environments (reception centre for youth and the specialised orphanages). However, when controlling for the outcome of the asylum process, which included assessment of age and denial or asylum, these effects were not significant, suggesting that the asylum process in itself has a detrimental effect on mental health, regardless of placement type. None of the refugees in this study received any form of intervention or support for their mental health. Similarly, high supportive care arrangements were associated with lower depressive and PTSD scores amongst 78 UASC from the Balkans and Africa in the UK, even when controlling for number of traumatic experiences or types of trauma experienced on a self-reported trauma scale (Hodes et al., 2008). In particular, care arrangements that involved 24 hour supervision such as foster care or small living groups were associated with less reported psychological distress than large reception centres in a study from the Netherlands (Bean et al., 2007). Another study, looking at 304 Sudanese UASC in the USA, found that those placed in same ethnic group living arrangements reported less PTSD and depressive symptoms on self-report scales (Geltman et al., 2005). This was also highlighted in a systematic review looking at care arrangements in UASC, whereby culturally matched families were associated with increased mental health resilience (O’Higgins et al., 2018). Taken together these findings highlight the importance of nurturing and culturally sensitive care arrangements.

### Summary of Literature on Resilience in UASC

#### Parallels and Differences of Past and Present and Across Cultures of UASC Populations

Despite significant limitations, when including assessment and perspectives of what helped and hindered recovery and acculturation in refugee groups across time, contexts and cultures, common themes emerge both in quantitative assessment of RFs and the accumulative testimony of survivors. These are distributed over multiple levels (see Table 1). In terms of contextual factors within the host country, the different groups of children reflect the importance of positive, nurturing, and less restrictive care arrangements (Bean et al., 2007; Freud & Dann, 1951; Klein, 1974), ideally in environments with cultural overlap (O’Higgins et al., 2018). The Windermere Children were all placed in a large hostel in Windermere, under the care of Oscar Friedman, a pre-War German refugee and psychologist, whose approach to care was hugely successful (Smith, 2008). As described retrospectively in the accounts of survivors, there were few simple rules, and the focus was on entertainment, sport, and education, which focused on Jewish history and religion, and the English language, history, and current affairs (Kushner, 2010; Smith, 2008). The importance of relationships with peers who had shared similar migration experiences also emerged across time in terms of being able to share feelings in age specific terms, maintaining connection to own culture within the host country and as cultural ‘bridge builders’. These relationships may be lifelong. For example, Smith (2008) describes how a club opened in London for social activity and sports, which many Holocaust refugee survivors even decades later referred to as a family and home (Smith, 2008). In the more recent groups of refugees seeking asylum throughout Europe, the importance of continuity of contact with family members in the home country and integration of the past and the present as part of the acculturation process, and development of their new identities also emerged as a theme. Data on all groups reported the importance of sharing positive memories about their pre-migration experiences and nurturing past relationships as a way of fostering resilience.

**Table. 1:** Resources and resiliencies to support recovery and integration amongst UASC

1	**Individual factors**
1.1	Coping and problem-solving skills
1.2	Personality factors e.g. easy temperament, humour, pro-social behaviours
1.3	Embracing independence
1.4	Forming meaning from their experiences
1.5	Chameleon-like talent - appearing ordinary and normal
1.6	Faith in a higher power or religious orientation
2	**Relationships**
2.1	*Families and attachments*
2.1a	Physical or virtual contact with families/extended families
2.1b	Connectedness of family prior to migration
2.1c	Close attachment to other adults e.g. caregiver, guardian, teacher, social worker
2.1d	Appeasing uncertainties to loved ones being dead/hurt/disappeared
2.1e	Facilitating continuity of experience and connectedness with culture of origin
2.2	*Role of peers*
2.2a	Support from peers and adults from similar cultural backgrounds
2.2b	Forming relationships with peers with similar flight experiences
2.2c	Forming relationships with peers/mentors at school
2.2d	Positive relationship with prosocial institutions e.g. schools or churches
3	**Process of acculturation: time and continuity**
3.1	Time spent in the country
3.2	Maintaining aspects of their culture of origin e.g. religion, people from own culture
3.3	Embracing aspects of local culture e.g. language, styles of dressing…
4	**Care arrangements**
4.1	Supportive living arrangements e.g. foster care or small living groups vs reception centres or restrictive environments
4.2	Placements promoting freedom with few restrictions
4.3	Experiencing a positive asylum and age assessment process
4.4	Living with culturally matched families

Overall, pre-migration factors and various post-migration factors are likely to operate together and interact with each other across many levels. Both pre- and post-migration factors appear to contribute to the mental health trajectory of UASC (Keles et al., 2018), and acculturation hassles appear to predict mental health problems independently of general hassles (also experienced by other youngsters) (Keles et al., 2016). However, the impact of the post-migration environment on increased resilience and healthier outcomes, including the positive effects of social support, cultural continuity and acquisition of competence in the new culture (Oppedal & Idsoe, 2015), has clearly emerged over decades of study and personal testimony. The implications for intervention and policy will be discussed below.

## Discussion

UASC are the most vulnerable group among refugees. A large proportion of UASC have experienced extreme levels of trauma and/or exploitation, including modern time slavery or being drafted by military groups as child soldiers. On arrival, they must negotiate the acculturation and postflight challenges including protracted uncertainties with regards to their residential rights, all without trusted adults. Unsurprisingly, prevalence of mental health problems is extremely high. Though there is insufficient provision of CAMHS, even where interventions are available, engagement has been an almost universal challenge for this population. While there are multiple reasons for this, these difficulties suggest that new approaches to engagement strategies and service provision need exploring. Considering the significant stigma attached to seeking mental health support, fostering resilience might be a promising avenue for adjusting the focus for MHPSS for UASC. In order to guide our hypotheses more specifically we explored strategies that might promote resilience in UASC. We considered current evidence relating to resilience in adolescents in general, and factors promoting resilience in UASC specifically. We included qualitative assessments of UASC individuals’ own current views and retrospective perspectives of what has helped during their postflight periods.

Resilience factors are distributed across a multilevel framework comprising intra-individual, and systemic micro- and macro-ecological factors. General RFs on an intra-personal level which appeared shared across all refugee and non-refugee groups are cognitive flexibility, problem-solving skills, and a stable self-concept. The latter, however, seemed to be promoted in refugee groups by fostering the bi-cultural identity and integration of past and present narratives. Lifetime relationships constituted an important systemic factor. Secure attachment and positive family and peer relationships appeared beneficial across all groups. In UASC it affected postflight recovery in multiple specific ways. Treasuring the happy memories of their past and family was reported as a resource of strength and happiness. Positive past relationships predicted maintaining connections with family members in their homeland and these were associated with positive relationship building in their new environments. Peer relationships played a specific role in UASC as it provided opportunities to share lived experiences, and facilitated engagement in cultural activities such as religious practice, social hubs, or speaking their home language. Peers also appeared to act as ‘bridge builders’ to the new cultures. Interestingly, positive acculturation appeared to include maintaining the connection to the old- as well as adoption of new cultural practices. Appearing ordinary and normal, adopting local habits, building new relationships and engaging in local culture via prosocial institutions like schools or churches equally feature as RFs for UASC alongside treasuring their roots. In addition, UASCS appear to draw resilience from nurturing homes and care arrangements that allow high independence and self-agency. In summary, our main findings highlight the importance of systemic resilience factors distributed over various levels of social connectedness, as well as narratives that integrated social connections and cultural values longitudinally and cross-sectionally. In UASCs, this specifically included lifetime relationships, care arrangements and acculturation in the host-country. Experience and quality of early life relationships, maintaining contact with families postflight, as well as forming relationships postflight with individual caregivers or peers from the same and different cultures, all seem to promote resilience in UASC. These appear alongside intra-individual factors, including embracing a bi-cultural identity.

The capacity to maintain and form positive relationships, highlights an important difference between UASC, and severely traumatised young people with impaired relational capacity due to attachment trauma. Psychological consequences of multiple traumatic experiences, where children became subjected to systematic and organised crimes or war, differs from psychological sequelae of early life adversities perpetrated by attachment figures (e.g. a parent or carer) (Fritz et al., 2018). This distinction is important when developing therapeutic interventions targeted to UASC.

### Implications for Practice

Our review suggests that intra-individual factors and inter-individual factors that promote relatedness, connectedness, and relationships are central to resilience amongst UASC. These include lifetime relationships, acculturation. In many ways this is unsurprising in a group that has been uprooted and displaced from their own cultural environments in addition to having lost their immediate supportive network of the family environment, during a critical developmental period that is programmed for social connectedness (Chase et al., 2008; Demazure et al., 2018; Ehntholt & Yule, 2006; Mels et al., 2008; Sierau et al., 2019; Sleijpen et al., 2016; Sujoldžić et al., 2003). If social connectedness is the most urgently perceived need and the best predictor of recovery, perhaps this needs to be addressed as an essential component in any therapy program. A focus on social and cultural connections may reduce the focus on pathology and adversity that, in the minds of UASC, is so heavily stigmatised. Social connectedness is on its own associated with better outcomes (Chase et al., 2008; Demazure et al., 2018; Ehntholt & Yule, 2006; Mels et al., 2008; Sierau et al., 2019; Sleijpen et al., 2016; Sujoldžić et al., 2003) and its emphasis may promote engagement prior to trauma reprocessing work. Interventions might therefore focus on enhancing the cross-cultural social network of the individual. This may comprise promoting connectedness with positive relationships in the home- and host country, life-history work that develops narratives that include past and new experiences with a focus on resource-building experiences in addition to trauma, and promoting integration of old and new cultural values. The journeys of UASC already highlight the resources that are present in those individuals, as these have enabled them, despite trauma, to leave their countries and successfully negotiate travel across long and potentially dangerous routes, and then to begin to engage in the resettlement and acculturation process without the company of their families (Keles et al., 2018). Therefore, supporting their high levels of self-agency might also be a necessary component in the therapeutic design. This may span from advocacy for supported but independent living arrangements, involvement in decision-making, promotion of language acquisition, as well as gaining occupational and other skills that support self-sustenance and independent navigation of life in a host country, but also emphasis on exploration, discovery and choice in therapeutic interactions with as little as possible didactic elements.

### Gaps in Research and Future Directions

The review is necessarily limited by the scarcity of literature. The larger body of evidence is derived from quantitative studies where researchers had explored specific factors e.g. relationships, which might mean conclusions were biased by the *a priori* hypotheses of researchers. While some factors also feature in qualitative research and survivors’ narratives, these data are even more limited. Exploring the narratives of young people may also elicit attention to RFs, that are only evident from the eye of the beholder. Expanding research to include more qualitative aspects on subjective experiences of the individual UASC may shift away from a top-down trauma hypothesis driven approach and engender a more participatory, user informed development of interventions for this complex and highly vulnerable group.

## Supplementary Information

Below is the link to the electronic supplementary material.

Supplementary file1 (DOCX 26 KB)
