# Supplementary material for: Survivors of Hell: Resilience Amongst Unaccompanied Minor Refugees and Implications for Treatment- a Narrative Review
Source: J Child Adolesc Trauma. 2021 Jul 22;14(4):559–69. doi: 10.1007/s40653-021-00385-7 (PMC8586295; doi:10.1007/s40653-021-00385-7)
Supplement: Supplementary file 1 — Supplementary file1 (DOCX 26 KB) [file 40653_2021_385_MOESM1_ESM.docx]

**Supplementary Materials**

**Research method strategy used**

Inclusion and exclusion criteria

In all languages published observational studies including children and adolescents (under age 18) who entered a host country unaccompanied seeking asylum (considered to be refugees) and exposed to variables hypothesised to impact mental health outcomes were required.

Need to have specified sample size, demographics, nature, intensity of intervention, and outcome measures. Eligible if examined any treatment or non-treatment influences on mental health or psychological resilience in UASC. Interested in both quantitative and qualitative studies. Excluded if it did not specify whether asylum seeking refugee minors were accompanied or not.

Search strategy

Terms

- Unaccompanied
- Refugee/migrat- or asylum-seeking/er
- Child/adolescent/minor
- Mental health/psych-/resilienc*

Databases searched in May 2020

PROQUEST: 181 papers (abstract): ab(unaccompanied) AND ab(refugee OR migrat* OR asylum-seek*) AND ab(child OR adolescent OR minor) AND ab("mental health" OR psych OR resilien*)

- PROQUEST MEDLine 1946-current
- PROQUEST PsychINFO 1946-current
- PROQUEST Psycharticles 1946-current
- 107 screening title
- 59 screening abstract
  - Of which 41 were duplicates
  - 18 were new

PUBMED 1946-current: generated 116 papers: (((unaccompanied) AND (refugee OR migrat* OR asylum-seek*)) AND (child OR adolescent OR minor)) AND ("mental health" OR psych OR resilien*)

- 76 screening title
- 51 screening abstract

The reference lists of significant review papers and those identified as relevant papers were also searched for additional studies

TOTAL 59 relevant papers from the database searches

Selection of studies

Search results were combined and duplicates removed. Initial screening of titles and those not relevant were excluded. Abstracts then screened. Then read in full and final list of studies were included in this narrative review. The process was done by IMR with supervision by VD.
